# Automatic segmentation of lower limb muscles from MR images of post-menopausal women based on deep learning and data augmentation

**DOI:** 10.1371/journal.pone.0299099

**Published:** 2024-04-02

**Authors:** William H. Henson, Xinshan Li, Zhicheng Lin, Lingzhong Guo, Claudia Mazzá, Enrico Dall’Ara

**Affiliations:** 1 Department of Mechanical Engineering, The University of Sheffield, Sheffield, United Kingdom; 2 INSIGNEO Institute for in silico Medicine, The University of Sheffield, Sheffield, United Kingdom; 3 Department of Automatic Control and Systems Engineering, The University of Sheffield, Sheffield, United Kingdom; 4 Division of Clinical Medicine, The University of Sheffield, Sheffield, United Kingdom; University of Illinois Urbana-Champaign, UNITED STATES

## Abstract

Individual muscle segmentation is the process of partitioning medical images into regions representing each muscle. It can be used to isolate spatially structured quantitative muscle characteristics, such as volume, geometry, and the level of fat infiltration. These features are pivotal to measuring the state of muscle functional health and in tracking the response of the body to musculoskeletal and neuromusculoskeletal disorders. The gold standard approach to perform muscle segmentation requires manual processing of large numbers of images and is associated with significant operator repeatability issues and high time requirements. Deep learning-based techniques have been recently suggested to be capable of automating the process, which would catalyse research into the effects of musculoskeletal disorders on the muscular system. In this study, three convolutional neural networks were explored in their capacity to automatically segment twenty-three lower limb muscles from the hips, thigh, and calves from magnetic resonance images. The three neural networks (UNet, Attention UNet, and a novel Spatial Channel UNet) were trained independently with augmented images to segment 6 subjects and were able to segment the muscles with an average Relative Volume Error (RVE) between -8.6% and 2.9%, average Dice Similarity Coefficient (DSC) between 0.70 and 0.84, and average Hausdorff Distance (HD) between 12.2 and 46.5 mm, with performance dependent on both the subject and the network used. The trained convolutional neural networks designed, and data used in this study are openly available for use, either through re-training for other medical images, or application to automatically segment new T1-weighted lower limb magnetic resonance images captured with similar acquisition parameters.

## Introduction

Semantic segmentation of medical images enables quantitative analysis of tissue groups within the body *in vivo*. Spatially structured anatomical details can be gathered through medical image segmentation, facilitating scientific advancements in the analysis of musculoskeletal tissue disorders. For example, image segmentation has been used as a potential method to inform screening processes for muscle disorders such as sarcopenia [1], to characterise the structural responses of muscles to cerebral palsy [2] and myopathy [3] and could be used in future to analyse response to treatments [4]. With advances in image analysis, methods to automatically isolate structural muscle characteristics might become available.

Deep learning is the branch of Artificial Intelligence (AI) that is currently used in state-of-the-art image analysis applications, so named due to the large number of connected layers used to isolate key features from within images [5,6]. These computational architectures have been proven powerful in the segmentation of several tissues from medical images, such as cardiac tissue from Magnetic Resonance Images (MRI) (average Dice Similarity Coefficient (DSC) calculated by comparison of automatic and manual segmentation of 0.93, [7]), brain tissue such as the hippocampus from T1-weighted MRI (DSC averaging 0.89, [8]), and the lungs from hyperpolarized MRI (DSC averaging 0.94, [9]). Moreover, due to a broader availability of computational processing power and advancements in graphic processing units, these tools are quickly becoming an important method for automatic image segmentation across many different applications. For example, since 2015, upon publication of the paper that first introduced the convolutional neural network (CNN) algorithm, “UNet”, developed for biomedical image segmentation [5], the number of research articles using UNet or its variations to segment medical images has dramatically increased [10]. The current consensus in the literature is that this base network architecture is still state-of-the-art, as demonstrated by the results of the Medical Segmentation Decathlon, set in 2022 [11]. The challenge consisted of identifying one generalized solution to the problem of medical image segmentation, including ten tissues/organs (brain tissue, prostate, lung, abdominal organs, heart tissues, lesions, cell nuclei, and others) within the human body, captured with different imaging modalities. Almost all methods submitted were able to segment all of the 54 imaging databases included in the challenge, with the best performing models employing CNNs [12]. These results suggest that CNNs, and more specifically, UNet style approaches might offer the best current solution for medical image segmentation.

Muscle segmentation from MRI, however, was not among the challenges submitted in the Medical Image Segmentation Decathlon. Through the segmentation of muscle tissue from medical images, muscle properties such as volume, length, and level of fatty infiltration could become measurable [13]. These properties are pivotal in muscle function and can be indicators of the structural health of individual muscles, muscle groups, or the entire muscle body [14–16]. Muscle segmentation was not included in the challenges submitted, however, there are many studies in the literature that have sought to segment muscle tissues from medical images. Most currently available approaches focus on segmenting the muscle tissue from the other tissues visible within the medical images (e.g., fat, bone, or intra-muscular fat) [15,17,18]. While such methods can be useful in assessing the total muscle volume or general level of fat infiltration of the muscle tissues, they provide no indication of specific muscles that may be subject to atrophy, nor do they highlight muscles that are smaller than their neighbours and could be the reason for an unstable joint. Not only are these muscle specific properties key in identifying and diagnosing muscle disorders, but also, subject specific muscle characteristics are also used widely to populate biomechanical models with muscle specific contributions to motion [19,20]. Such models have been used extensively to isolate the muscles that limit motion, and measure pre-operative versus post-operative performance [21]. These models can be particularly powerful when assessing the risk of fall of older individuals, particularly post-menopausal women, who have a higher chance of developing osteoporotic fractures [22].

Nevertheless, muscle segmentation using CNN approaches applied to MRI is challenging because there are few openly available datasets that would be large enough to facilitate learning-based methods, and that the available datasets are inhomogeneous in their acquisition parameters [23]. Other segmentation techniques using deformable registration and statistical shape modelling have been explored but showed only limited accuracy (DSC in the range of 0.90–0.99 for groups of muscles, and 0.40–0.87 for single muscles) can be achieved [24,25]. For example, in a previous study, the use of a single and multi-atlas image registration algorithm (the Sheffield Image Registration Toolkit, or ShIRT [26]) was explored for automatic muscle segmentation of a subset (N = 5) of the study cohort used in this paper. The registration-based methods used in this previous study were able to output segmentation of average Relative Volume Error (RVE) of -2.2%, average DSC of 0.73, and Hausdorff Distance (HD) of 25 mm. Only a few studies have used deep learning techniques to perform muscle segmentation from MRI. Ding et al. segmented seven MRI datasets (water and fat suppressed images were acquired from the thigh, voxel size = 0.75 x 0.75 x 6mm^3^) using a standard UNet architecture trained on 23 datasets (3:1 split in training to validation, 19 healthy and 4 patients with neuromuscular diseases) [13]. This group segmented two muscle groups (knee extensors and flexors) and individual muscles (sartorius and gracilis) from multi-acquisition with a mean DSC of 0.90±0.03 and 0.86±0.04, respectively, suggesting that the segmentation of individual muscles is more challenging than muscle groups. In another study, Zhu et al. built on that work and investigated whether adjustments to the network architecture would yield individual muscle segmentations of improved accuracy [2]. Zhu et al. [2] segmented 13 muscles from the shank of 4 children using T1-weighted images, with 15 subjects used for training and 1 used for validation (age range: 5.4 to 14.8 years old, 6 females, 14 males, 6 with cerebral palsy). In this study, the original UNet and the best performing variant of it presented similar accuracy (0.87±0.01 and 0.89±0.00 in DSC, respectively). However, neither study sought to segment all individual muscles from lower limb MRI. Ni et al. used UNet to segment all lower limb muscles of a testing dataset of 13 young healthy adults, using 51 subjects for training the network (training and validation split not stated) [27]. In this study, an individual network was trained for each of 35 muscles, each consisting of two neural networks trained in series: the first to crop the lower limb three dimensional (3D) in order to isolate only the muscle being segmented, and the second to segment the muscle from the cropped image. The accuracy of the segmentations was comparable to that achieved with manual segmentation methods for 14 of the muscles, and slightly worse in the other 21, with 0.90±0.06 DSC across the 35 muscles segmented [27]. From such results, it can be concluded that learning-based methods have big potential in accurately segmenting individual muscles or muscle groups, if trained on reasonably sized cohorts. However, acquiring large MR imaging datasets is often unrealistic due to the high associated cost. Therefore, methods to reduce the burden of requirement of these large databases should be explored.

**Fig 1:**
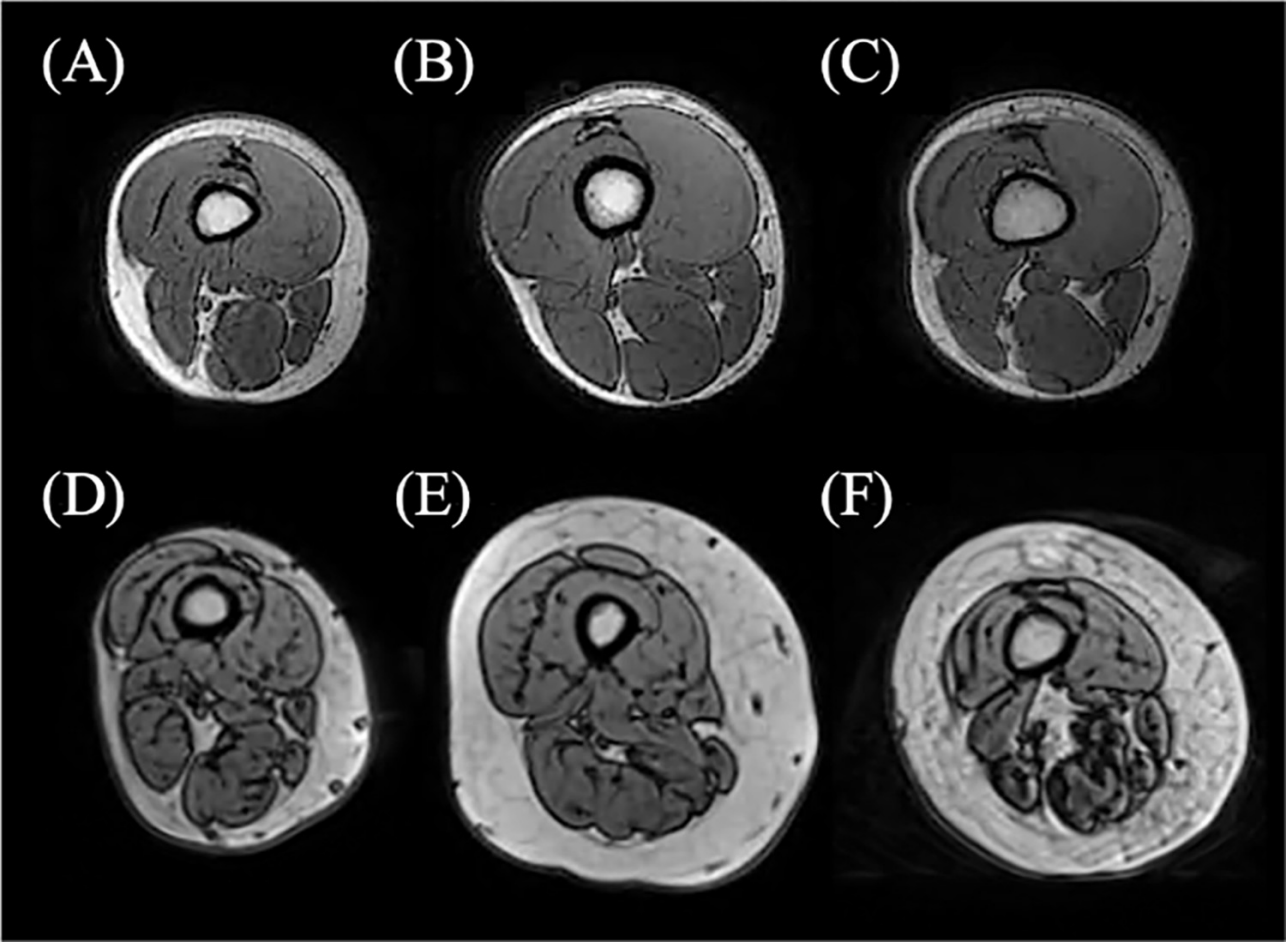
Comparison of thigh MRI for young healthy individuals (A, B, C) and older individuals (D, E, F).

While the above studies highlighted the potential of deep learning approaches for muscle segmentation, there are some notable limitations. Firstly, individual muscles from MRI of children and healthy subjects have been explored, but, to the authors’ knowledge, their application to segment individual muscles of postmenopausal women has not been demonstrated yet. Segmenting muscles from individuals at risk of age-related muscle degeneration (e.g., loss of muscle mass and strength in the case of sarcopenia) presents a complexity not previously addressed for deep learning-based muscle segmentation methods [4], as shown in Fig 1. Secondly, the above-mentioned approaches have used variants of standard network architectures to segment portions of the lower limbs or muscle groups, which may not be ideal when used to simultaneously segment single muscles from different parts of the whole lower limbs. Attention channels could be used to overcome this limitation, as they allow the spatial location of elements within each image to be incorporated into the learning process, and these have been used to boost accuracy in the segmentation of several tissue groups, such as brain tumours [28], glands [29], and liver [30]. Attention as a mechanism has not been extensively used in the context of muscle segmentation but was previously reported by Zhu et al. [31] to boost the segmentation capacity of a modified UNet architecture. This study explored the use of the attention mechanism to segment 11 muscles and 2 bones, and its inclusion boosted segmentation accuracy from 0.82 DSC to 0.85. Therefore, the attention mechanism might offer a similar boost to performance when used to segment upper and lower leg muscles simultaneously. Additionally, referencing the spatial meta-data (where along the lower limb an image was acquired from, for example) that is captured simultaneously with all medical images could be incorporated into the learning process, but has not been explored for muscle segmentation. Finally, the requirements of large databases for good model performance are not readily available and as such, methods to use available data to the fullest extent should be explored.

Therefore, the aim of this study was to evaluate the accuracy of the state-of-the-art (UNet [5] and Attention UNet [32]) and a novel (“Spatial channel UNet”, accounting for spatial meta-data) CNNs to segment individual muscles of the lower limbs from T1-weighted MRI of postmenopausal women. Secondly, we investigated the effect of bolstering the training process of each architecture with augmented images, to assess whether the need for large, fully segmented databases to train learning-based automatic segmentation methods can be alleviated.

## Methods

### Data acquisition

#### Participants and image acquisition

Retrospective T1-weighted MRI of the lower limb from 11 post-menopausal women (mean ±standard deviation: 69±7 years old, 66.9±7.7 kg, 159±3 cm) were used for this study [33,34]. Images were collected using a Magnetom Avanto 1.5T scanner (Siemens, Erlangen Germany), with an echo time of 2.59 ms, repetition time of 7.64 ms, flip angle of 10 degrees. The study was approved by the East of England–Cambridgeshire and Hertfordshire Research Ethics Committee and the Health Research Authority (16/EE/0049). The MRI were acquired in four sequences, capturing the hip, thigh, knee, and shank, respectively. To reduce scanning time while still providing detailed geometries of the joints (requirement for the original study for which this dataset was acquired [33], the joints were acquired with a higher resolution (pixel size 1.05 mm^2^, slice spacing 3.00 mm) than the long bone sections (pixel size 1.15 mm^2^, slice spacing 5.00 mm).

#### Manual segmentation and label generation

**Fig 2:**
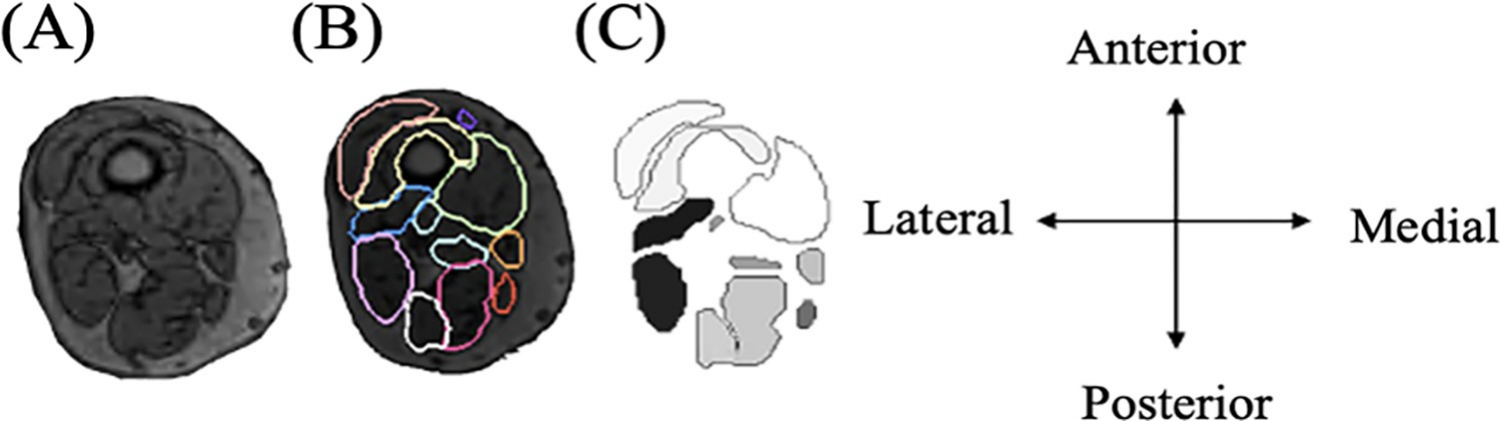
The process of generating the inputs for the neural network. The MRI (example thigh 2D slice) inputted into the neural network (A) was manually segmented (B) and the segmentations were transformed into a labelled image (C). The greyscale colours of each muscle were ordered from 1–37 as they appear alphabetically.

The T1-weighted image sequences were stacked in MATLAB to form one continuous 3D image from hip to ankle, by homogenising the resolution of each of the imaging sequences taken from the different sections to be 1.00x1.00x1.00 mm^3^ through tri-linear interpolation (interp3, MATLAB 2006a). The fields of view of the images across the four sequences were equated by wrapping the images in blank data (greyscale value of 0) and referencing the spatial metadata of the images to retain the relative subject position across the imaging sequences for each subject. Once stacked, the 37 muscles (named in Table 1), within the lower limb T1-weighted scans were segmented using the current gold standard approach, manual segmentation, using Materialise Mimics [35]. The output of the manual segmentation procedure for each muscle was a 3D mask containing only the muscle tissue. For visualising the different muscles in the same image slice, the outputted binary image for each muscle was assigned a class (numbered 1–37) (Fig 2). Both limbs were included in the training and testing databases, with a reflection applied to the left limb to homogenise the imaging data. The CNNs were trained with all 37 muscles included, ensuring that predicted muscle tissue is more realistically distributed. The intra-operator reproducibility of the manual approach was tested in a previously published study [34]. In the previous study, one operator segmented all muscles from within one subject three times and the muscle volumes found through these repeated segmentations were used to calculate the Coefficient of Variation (CoV) of the muscle volumes [34]. Following literature suggestions [4,33,34], the muscles for which the CoV of the muscle volumes exceeded 10% were removed from the study. In addition, the previously published study excluded the muscles for which the external borders either laid outside the field of view or those that were not clearly visible from within the images [34]. Therefore, 23 muscles were deemed able to be segmented reliably, in context of the intra-operator repeatability analysis on the current gold standard manual segmentation approach, and those that were not (14 muscles) were removed from the analysis of results as they would incur bias. All muscles included in the training are noted in Table 1, and those with a noted colour (the colour they appear in the results section) are those included in the analysis of results (i.e., the 23 muscles that can be reliably segmented manually).

**Table 1:** The 37 muscles included in the training phase for each network, separated into the areas of the body. The 23 muscles highlighted in bold were included in the analysis of results, with those that are not highlighted being excluded in response to the inclusion criterion outlined above. The abbreviations associated with the included muscles are used in the results section to identify the corresponding muscles.

Body Section	Muscle	Colour
Hips	**adductor brevis**	AB
	**adductor longus**	AL
	**adductor magnus**	AM
	gemellus superior	N/A
	**gluteus maximus**	GLM
	gluteus medius	N/A
	gluteus minimus	N/A
	**Iliacus**	IL
	obturator externus	N/A
	obturator internus	N/A
	Pectineus	N/A
	Piriformis	N/A
	Psoas	N/A
	quadratus femoris	N/A
Thigh	**biceps femoris caput breve**	BFCB
	**biceps femoris caput longum**	BFCL
	**gracilis**	GR
	**rectus femoris**	RF
	**sartorius**	SA
	**semimembranosus**	SB
	**semitendinosus**	ST
	**tensor fasciae latae**	TFL
	**vastus intermedius**	VI
	**vastus lateralis**	VL
	**vastus medialis**	VM
Calf	extensor digitorum longus	N/A
	extensor hallucis longus	N/A
	flexor digitorum longus	N/A
	flexor hallucis longus	N/A
	**gastrocnemius lateralis**	GL
	**gastrocnemius medialis**	GM
	**peroneus brevis**	PB
	**peroneus longus**	PL
	popliteus	N/A
	**soleus**	S
	**tibialis anterior**	TA
	**tibialis posterior**	TP

### Augmented imaging data

The CNNs were trained using both the original MRI and a database of augmented images. These augmented images were generated in a previous study [25], using deformable image registration. Each of the 11 subjects were paired with one another and their associated MRI were input to the registration pipeline, using both subjects as both the fixed and the moving image. The 3D imaging data of the two subjects were registered, enforcing a deformable, non-linear registration. The result of the process was a new, non-linearly deformed set of imaging data, which, after applying the displacement vector field to the images containing the segmentation of the moving subject, was fully segmented, with accuracy identical to that of the manual process. Each of the 11 subjects were registered to one another, generating 110 anatomically distinct augmented imaging datasets. As described in [25], these subjects were vetted by experts in muscle segmentation according to a set of exclusion criteria requiring that the augmented subjects were anatomically feasible, after which the number of suitable 3D imaging datasets (or virtual subjects) of the lower limb was reduced to 69. In a previous study [34], the anatomical variability of the augmented database was compared to that of the original database, highlighting that the shape and size of the muscles within the augmented database were significantly different to those within the original database. In addition, some example inputs (fixed and moving images) and outputs (augmented images) of the registration procedure are shown in Supplementary 1 to highlight the anatomical diversity between original and augmented images.

### Convolutional neural networks

Three CNNs were used: the “UNet” [5], the “Attention UNet” (A-UNet) [32], and an in-house model, named “Spatial channel UNet” (SC-UNet). The first two network architectures are well-known, state-of-the-art architectures that have been extensively used throughout the field of medical image analysis. A-UNet has not yet been tested in the context of muscle segmentation to the best of the authors’ knowledge, hence its inclusion in this study. In the following sections, the structure and details of these network architectures are briefly explained.

### UNet

The UNet architecture is a 2D multi-layered deep CNN with encoding and decoding pathways. Within the UNet architecture, sequential convolution operations are applied firstly to the input image and thereafter to feature maps: the matrices that enable feature extraction [5]. The number of convolution operations, and therefore, the number of features channels extracted in each convolution layer followed those imposed by the original UNet architecture [5], being: 64, 128, 256, 512, and 1024 at each respective stage of the encoding path. Max pooling (with a 2x2 kernel) was used in this study, as it has been proven to be an efficient and effective method of pooling in medical image segmentation problems, such as cell segmentation [5], brain tissue segmentation [36], and many others [37]. Max pooling of a feature map passes the maximum element in each 2x2 block of the feature map to a downsampled feature map. The 2x2 kernel used in this study was applied with stride 2, reducing the size of the downsampled feature map to half the size of the input feature map. Batch normalisation was used between each convolution block, normalising the weights assigned to each feature map across each mini-batch passing through each stage of the network. The inclusion of batch normalization was found to increase the time taken to complete each epoch of training, but to reduce the overall number of epochs required to train a functional model [38]. Like the contracting path, each of the four stages of the expanding path consisted of one up-sampling block, followed by two convolution blocks. Additionally, feature maps from the contractile path with the same size were copied and concatenated (in a layer named skip-connections) after the up-convolution was applied, coupling the features found within the contractile path with the expanding path. The design of the expanding path was focused on discovering where the relevant features are in the image. When coupled with the contracting path, the UNet is capable of learning what and where it is looking in the images during the training process and using this understanding in the testing phase.

The final stage of the UNet architecture occurs once the feature map was up-sampled back to the size of the input image, i.e., after four down-sampling and four up-sampling stages. The final stage was a convolution layer where the number of feature channels was equal to the number of classes required to be segmented, in this case 37. The output was a probability map defined for each pixel in the input image, which was used to create a segmentation prediction, finding pixel-wise the class of greatest probability.

### A-UNet

The second model employed an attention module built directly into the traditional UNet architecture, following the theory proposed by Oktay et al. [32]. The attention encoder allowed the spatial location of the region of interest to be retained. The module added together the feature maps from the down-sampling and up-sampling stages, through multiplication of the two and then normalization. In this process, the important feature channels (those with the greatest weights) from both the down-sampled and up-sampled images were accentuated, and conversely de-emphasizing those feature channels that were not important. The attention gates consisted of: 1) a simple matrix addition, 2) a rectified linear unit, forcing feature channels of negative weights to be equal to 0, 3) a sigmoid function, to squeeze the weights to be in the range (0,1), and finally 4) a resampling stage, to retain the correct feature map and feature channel sizes, as described by Oktay et al. [32].

### SC-UNet

This model followed the CNN operations of the traditional UNet, with an additional spatial channel running in parallel, designed to enrich the network’s learning process with the knowledge of where the image slice was acquired from within the lower limbs (along the axial direction). This method builds an understanding into the network of what muscles could and should be segmented from within each image. For example, thigh muscles, such as the vastii, are never present within an image slice taken from the calf, and the addition of the spatial channel is designed to allow the network to use this understanding for unseen testing subjects.

**Fig 3:**
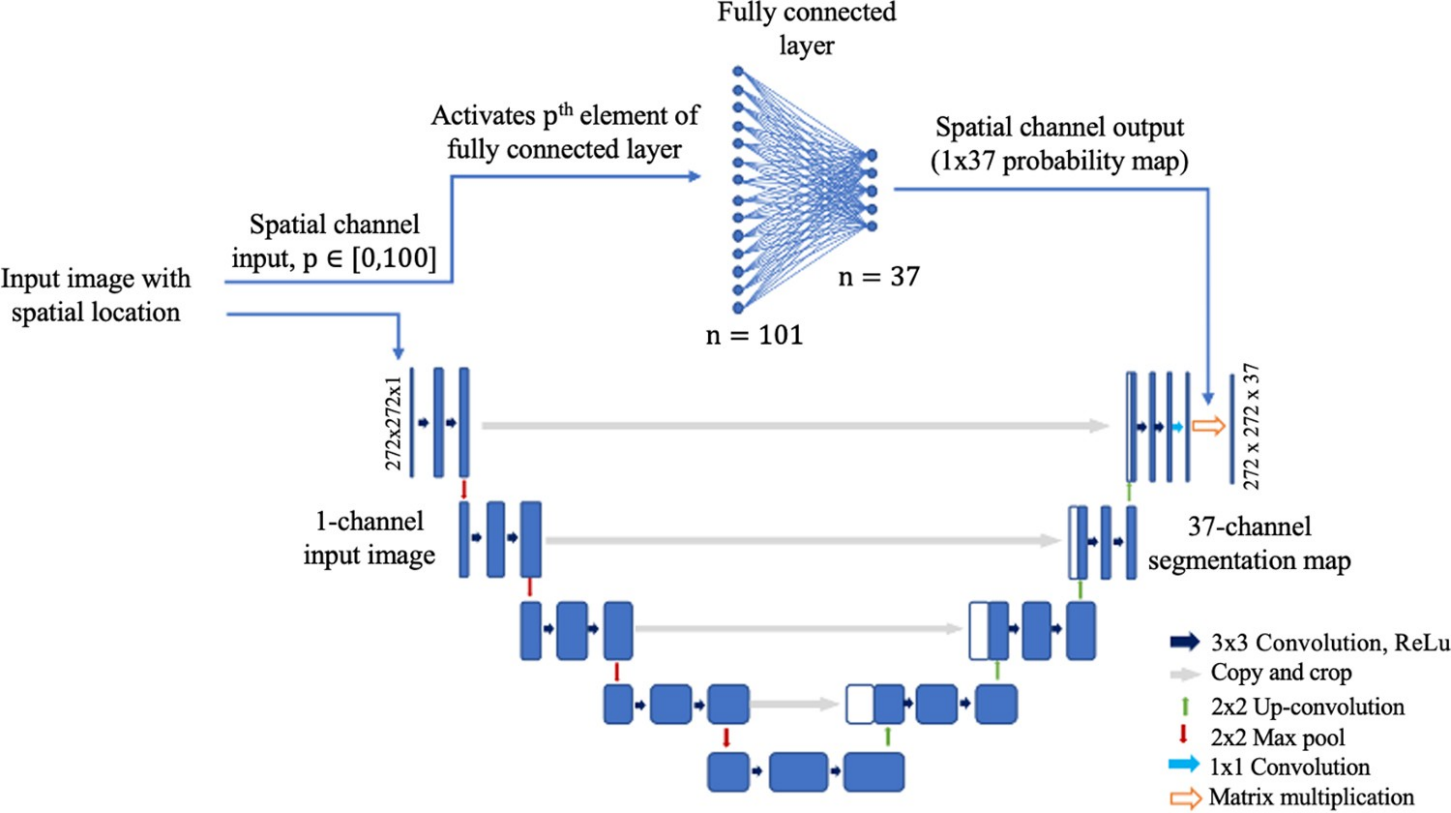
The Spatial channel UNet (SC-UNet). The spatial location of the input image was first calculated. Spatial information and the image were inputted into the network simultaneously, where they were split instantly. The image data went through the standard UNet architecture, while the spatial channel took an integer, *p*∈[0,100], and activated the p^th^ node of the input to the fully connected linear layer. Each input node was connected to each output node, of which there were 37 (equal to the number of muscles), allowing the locations of the muscles to be learned along the longitudinal axis.

The architecture of the SC-UNet (Fig 3), maintained the original UNet structure, with a fully connected linear layer running in parallel. The model required a measure of the position along the lower limb each image was acquired from, which was contained in the meta data of all images. The percentage along the lower limb (*p*) from which each image was acquired was calculated and used to provide this information, referencing the image number and the total number of images within each sequence. This spatial reference was tied to each image and both were simultaneously inputted into the network model. The MRI ran through the UNet portion of the model and the spatial reference ran through the fully connected linear layer, in parallel. The fully connected layer had 100 input neurons representing each percentage along the lower limb, and 37 output neurons representing the number of classes (individual muscles) being segmented from the images, with all input and output neurons being connected. The percentage along the axis of the lower limb (*p*) calculated for each image slice was converted into a 100x1 binary matrix, with the *p*^th^ element set equal to 1 and all other elements equal to 0. The network was trained to strengthen connections between a given position along the lower limb and the expected muscles that were contained in images acquired from those positions, and conversely, weaken the connections for those muscles that were not. The output of the spatial channel was a probability matrix of each muscle being within a certain image, which was then multiplied with the result of the final convolutional layer in the UNet structure, influencing the segmentation results.

### Training method

All three models were trained using identical protocols, on a NVIDIA® GeForce RTX™ 3060 Ti Graphics Processing Unit (GPU) with 8GB of memory. A stochastic gradient descent method was used, separating the training dataset into batches (of size 8 due to memory card size). The Adam optimizing algorithm [39] was employed to iteratively update network weights during back-propagation. The learning rate was set at 0.001 and decreased to 0.0005 after 30 epochs of training, and further to 0.0001 after another 30 epochs [2]. In total, networks were trained for 90 epochs. All models were trained with the multi-class cross entropy loss function [40]. Other hyper-parameters were tuned empirically for the optimal network performance, whilst ensuring the GPU memory and capacity were not exceeded.

Throughout all network training, an 80:20 split between training and validation data was used. The training data was used to fit the models to the lower limb MRI. The validation data was used to verify that the algorithm was learning as intended in response to the training method selected. Due to the small number of subjects in the study cohort, a ‘leave one out’ testing system was used. Each model was checked for convergence through analysis of the training and validation loss curves. For example, the labelled imaging data of ten subjects were used to train (eight subjects used for training, two for validation) each of the networks. The trained network was then used to segment the testing subject, allowing the segmentation accuracy to be assessed. Network training using augmented images followed a similar rule.

### Assessment of model accuracy

Three tests were performed:

1. One subject was segmented by the three CNN models trained with the original database, allowing the accuracy of the different CNNs in automatically segmenting individual muscles to be quantified.
2. The CNNs were retrained including the augmented images (generated through deformable image registration) [25] in the training dataset. The same subject as in test 1 was segmented allowing the effect of including the augmented images to be analysed.
3. The three CNNs were retrained five times, exchanging the testing subject used in test 1 for five other subjects. Test 3 allowed the robustness of each network architecture to be tested.

In test 2), all three networks were retrained with the augmented images included in the training dataset, using the training protocol outlined previously. Fifty-two labelled subjects were used in the training dataset, and 16 were used in the validation dataset. Those augmented images created from the registration of images involving the testing subject, whether it be as the fixed or target subject within the registration [26], were removed from the training and validation datasets to avoid bias during training.

In test 3), each network was retrained five times independently, using each of the five subjects as the testing dataset. These five subjects were selected from the original 11 fully labelled subjects. As a leave one out approach was used, each network was trained with each of the other subjects within the training dataset. To prevent biased predictions when varying the testing subject, the networks were retrained each time by adapting the training dataset such that the augmented images related to the new testing subject were removed. The number of subjects within the training and validation datasets for each of the experiments is presented in Table 2.

**Table 2:** Number of datasets used for test 2. The “Initial test” column reports the number of subjects (each subject contains approximately 1000 images) within the training and validation datasets for the initial testing subject used in test 2). Columns “Subject 1–5” represent the other 5 subjects for whom the networks were retrained in test 3). These 5 subjects were then used in turn as the testing subject. Note that these 5 subjects are different from the initial test subject used in test 2).

Datasets	Initial test	Subject 1	Subject 2	Subject 3	Subject 4	Subject 5
Total	66	65	64	64	67	64
Training	52	52	51	51	53	51
Validation	16	15	15	15	16	15

### Error metrics

To interpret the segmentation accuracy, three complementary error metrics were calculated for each automatically generated muscle segmentation. Firstly, the Relative Volume Error (RVE, %), was calculated according to Eq 1, as the percentage difference between the volume of the automatically generated segmentation (V_A_) and the corresponding reference segmentation, collected through the semi-automatic process (V_R_).

$$RVE=100\times \frac{V_{A}-V_{R}}{V_{R}}$$ Eq 1

Secondly, the DSC [41] was calculated according to Eq 2, for each muscle as double the intersection over union of the automatic (A) and reference (R) muscle segmentations.


$$DSC=\frac{2(A\cap R)}{\left|A|+|R\right|}$$ Eq 2

Thirdly, the Hausdorff Distance (HD, mm) was calculated as the maximum between 1) the minimum distance between any point located on the surface of the automatic segmentations (A) and their closest points (r) located on the reference segmentation; and 2) the minimum distance between any point located on the surface of the reference segmentations (R) and their closest points (a) located on the automatic muscle segmentations [42], as expressed in Eq 3.


HD=max{d(A,r),d(a,R)} Eq 3

### Statistics

The not-augmented training results from each of the network models (distributions of RVE, DSC, and HD for the 23 muscles segmented in the testing subject) were tested for normality using a Kolmogorov-Smirnov test, concluding that they were not normally distributed. Thereafter, the results from the three different CNN models (test 1) were tested for statistically significant differences using a Kruskal-Wallis one-way Analysis of Variance (KW-ANOVA).

After retraining the networks with the augmented images (test 2), a statistical test was conducted, comparing the results with and without the augmented images included in the training for each network structure independently, to analyse the effect of incorporating the augmented imaging dataset into the training phase. To do so, a Wilcoxon signed-rank test was used, after a Kolmogorov-Smirnov test concluded that the error metrics were not normally distributed.

Upon retraining each of the three CNN models for five other subjects (test 3), two statistical tests were conducted. First, the error metrics found for each of the models was tested independently using a KW-ANOVA, with the subject as the independent variable. This statistical test was conducted to assess the robustness of the models to changes in the testing subject. Then, the RVE, DSC, and HD for each subject were compared across the three models (the CNN model being the independent variable), using a KW-ANOVA test.

### Comparison of model performance with respect to manual segmentation inter-operator repeatability

The results of the test 3 were contextualised against the inter-operator repeatability evaluated between two operators. As stated in the section titled ‘Manual segmentation and label generation’, the intra-operator repeatability was tested in a previous study [34]. That study also presented the inter-operator repeatability of the muscle volumes gathered through manual segmentation. However, the inter-operator repeatability of manual segmentation was not quoted in terms of DSC or HD. Therefore, the inter-operator repeatability was retested here, and so the 23 muscles were manually segmented from three random subjects by two separate operators. The DSC and HD were calculated following Eqs 2 and 3 considering the two manual segmentations. The RVE was calculated following Eq 1 after taking the absolute value as the RVE was averaged across the three repeated segmentations from different subjects and could be skewed by the averaging of both over or under estimations for a single muscle. The averaged values of absolute RVE, DSC, and HD were calculated and compared to the results of test 3. Through these analyses, the accuracy of the automatic segmentations was compared directly to the repeatability of the manual process.

## Results

### Initial model evaluation

#### Training convergence

**Fig 4:**
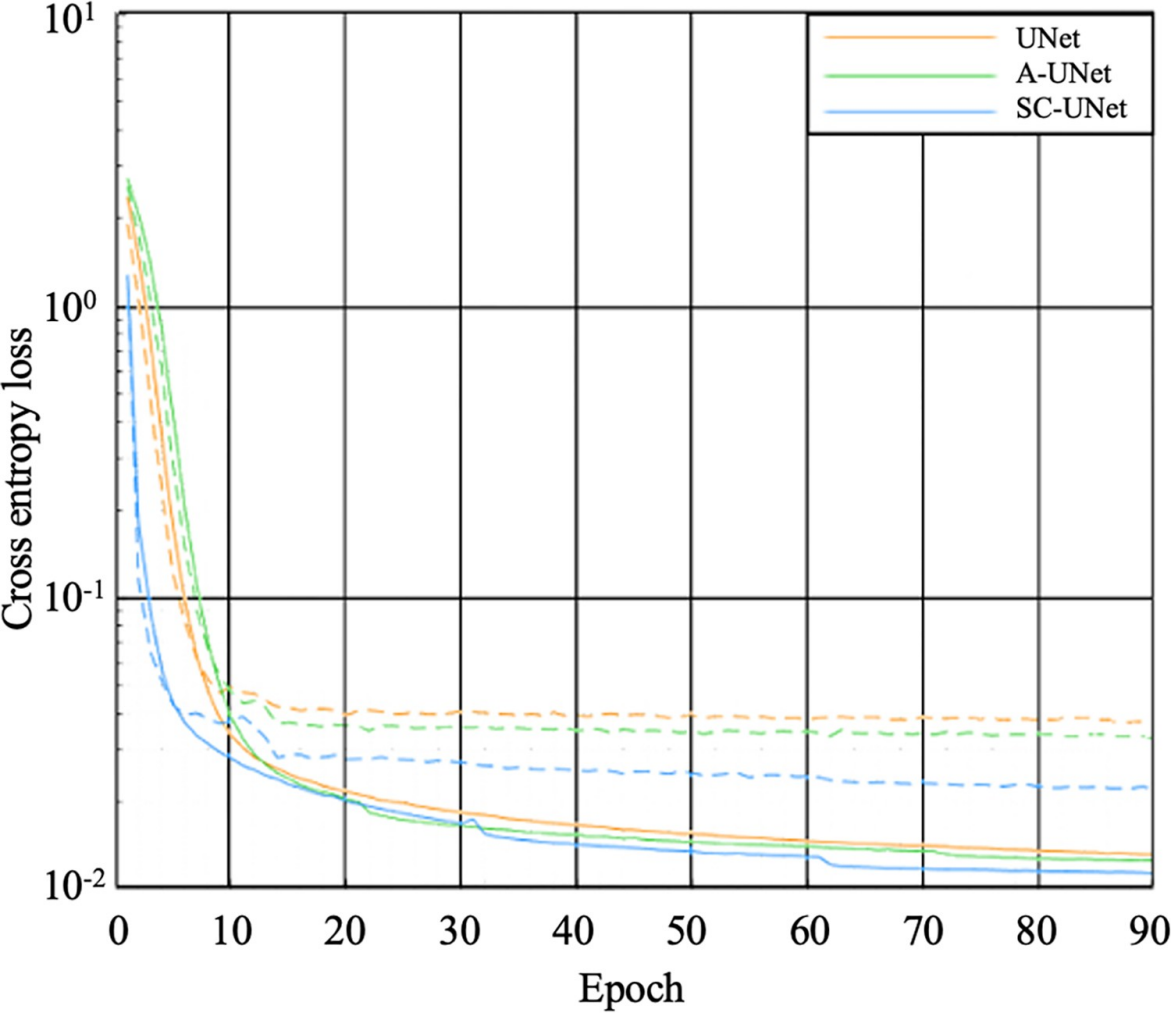
Training (solid) and validation (dashed) loss curves calculated throughout the training phase for the UNet (orange), A-UNet (green), and SC-UNet (blue). The cross-entropy loss shown was calculated for each batch of training or validation data and averaged across each epoch.

All models converged at the selected number of training epochs and each of the models were found to be a good fit to the training and validation data. For all three models, the training loss had converged at or before the 90^th^ epoch (Fig 4). Moreover, the validation loss was unchanged from the 30^th^ epoch, until the 90^th^ meaning that the parameters found for the models at the 90^th^ epoch were valid to use for the testing dataset.

### Number of parameters and training time

The details regarding number of parameters, time for each iteration, and overall training time are presented in Table 3. The UNet and SC-UNet had very similar numbers of parameters, while the A-UNet had a much larger number of them. The training time, reflected by the number of parameters involved, was comparable for UNet and SC-UNet, but the A-UNet required more time on average to train. The time to segment the testing dataset was less than 30 s for all models.

**Table 3:** Number of parameters, average number of batches trained per second, and total training time for the three CNN models tested.

	UNet	Attention UNet	Spatial channel UNet
Number of parameters	1745920	2444288	1749620
Average batches per second (it/s)	6.38	4.34	6.19
Total training time (hrs)	4.14	6.31	4.38

#### Initial segmentation accuracy

**Fig 5:**
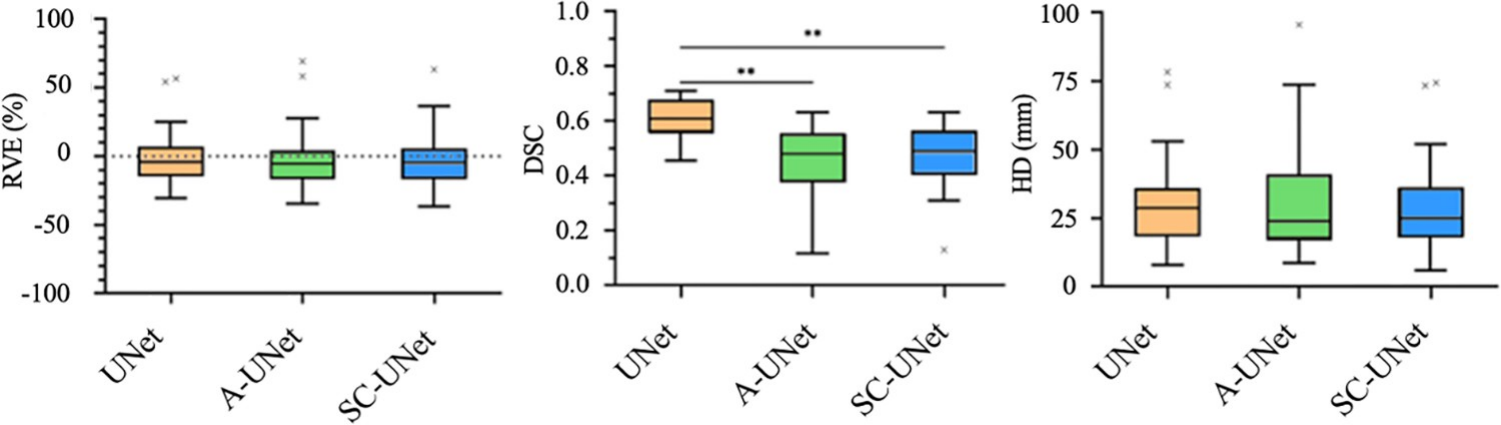
The RVE (%), DSC, and HD (mm) error metrics calculated through comparison of the reference and automatically generated segmentations for one testing subject across the three models tested (* p<0.05, ** p<0.01).

For the 23 muscles segmented, the RVE was comparable among each of the three networks, with no statistically significant difference found in the results (p>0.544, comparison of UNet and A-UNet, Fig 5). Specifically, the mean (± standard deviation) RVEs found for each of the three networks were -2.1±19.3% (UNet), -3.1±21.4% (A-UNet), and -3.5±18.2% (SC-UNet), with similar interquartile ranges.

The DSCs for the A-UNet (0.73±0.06) and SC-UNet (0.74±0.06) were not significantly different (p = 0.735), whereas the DSC found for the UNet (0.80±0.03) was significantly higher than the other two models (9.6% higher than A-UNet and 8.1% higher than SC-UNet, in both cases p<0.01) (Fig 5).

For HD, no significant differences (p>0.225, comparison between UNet and SC-UNet) were found between the means of the UNet (29.4±15.1 mm), A-UNet (30.9±19.0 mm), and SC-UNet (28.1±15.2 mm).

### Augmented data inclusion

**Fig 6:**
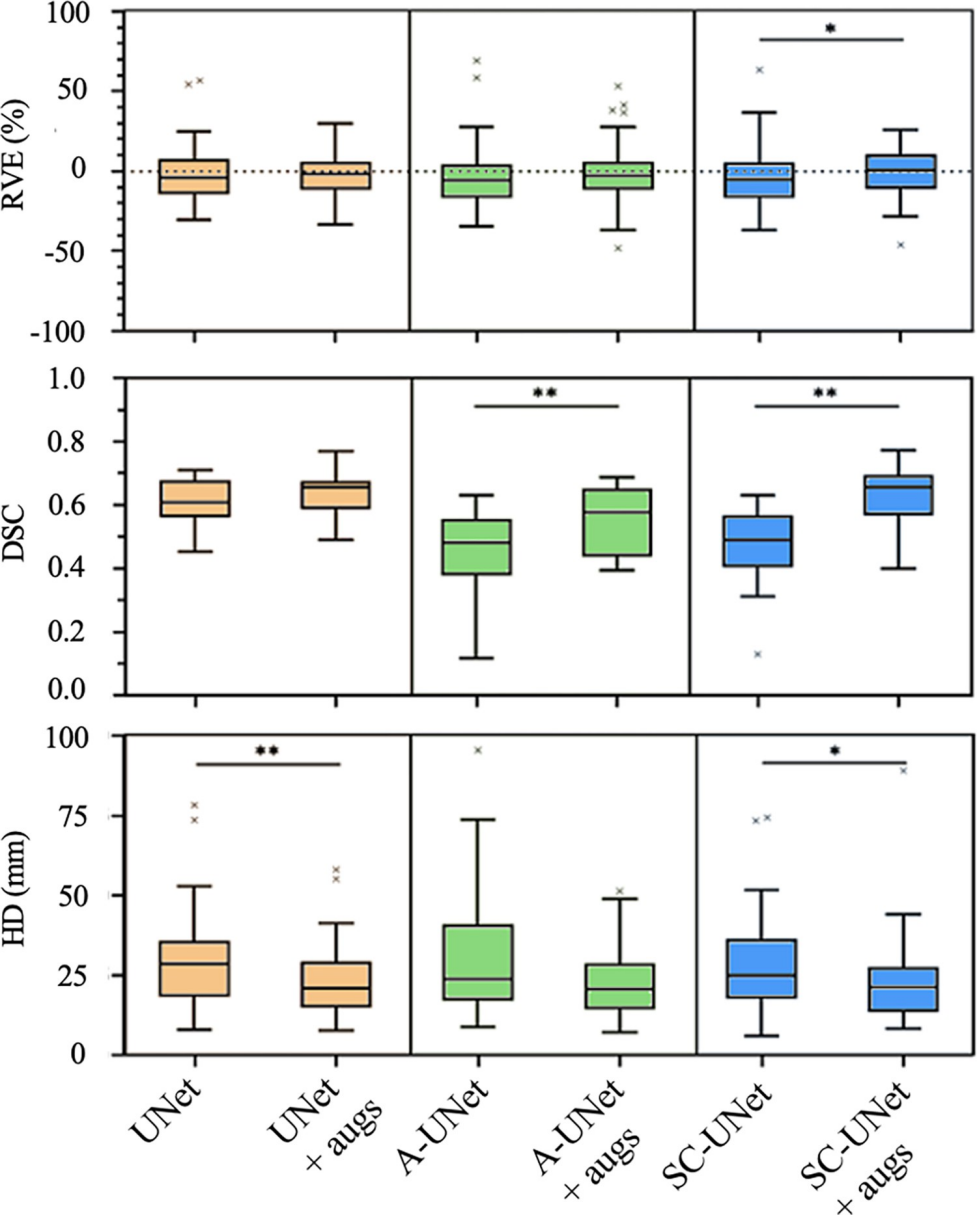
The RVE (%), DSC, and HD (mm) for the 23 muscles of the same testing subject as in Fig 5, calculated using each of the three CNN models tested, both with and without augmented images included in the training phase. Significant differences are reported with *(p<0.05) and ** (p<0.01).

Across all three error metrics, the 23 muscles of the test subject were consistently segmented more accurately after retraining the models with the augmented training datasets (Fig 6). The UNet improved the least after retraining, with no significant difference for RVE (p = 0.857) across the 23 muscles, but the upper and lower quartiles after inclusion of the augmented data fell within the acceptable level of operator variability of ±10% [34]. Similarly, the A-UNet showed no significant improvement in RVE between augmented and not augmented training datasets (p = 0.074). However, for SC-UNet, there was a small but significant reduction of RVE after training with the augmented subjects (from -3.5% to -0.5% RVE, p = 0.024).

Considering DSC, all three models were more accurate with the inclusion of the augmented subjects, with significant improvements for both the A-UNet (from 0.73±0.06 to 0.79±0.06, 8.2% increase, p = 0.007) and the SC-UNet (from 0.74±0.06 to 0.81±0.05, 9.5% increase, p<0.001). The DSC was improved drastically for those muscles segmented with the lowest DSC (gracilis, adductor brevis and peroneus longus) after inclusion of the augmented data, particularly for the A-UNet (from 0.56 to 0.72 for gracilis, from 0.66 to 0.80 for adductor brevis, from 0.66 to 0.77 for peroneus longus) and SC-UNet (from 0.57 to 0.72 for gracilis, from 0.65 to 0.86 for adductor brevis, from 0.67 to 0.76 for peroneus longus). The 1.4% increase in DSC for the UNet with augmented training datasets was not statistically significant (p = 0.879).

Training the model with augmented datasets decreased significantly the HD for the UNet (HD from 29.5±15.1 mm to 22.5±11.2 mm, 27.1% decrease, p<0.001) and the SC-UNet (HD from 28.1±15.2 mm to 22.8±13.7 mm, 18.9% decrease, p = 0.026). A similar but not-significant reduction of 16.1% in HD with augmented training dataset was found for A-UNet (p = 0.375). Overall, the traditional UNet trained with the augmented datasets segmented the test subject with the greatest accuracy, with consistently low RVE, high DSCs, and equivalent HDs compared to the other two CNN models.

### Retraining for different testing subjects

**Fig 7:**
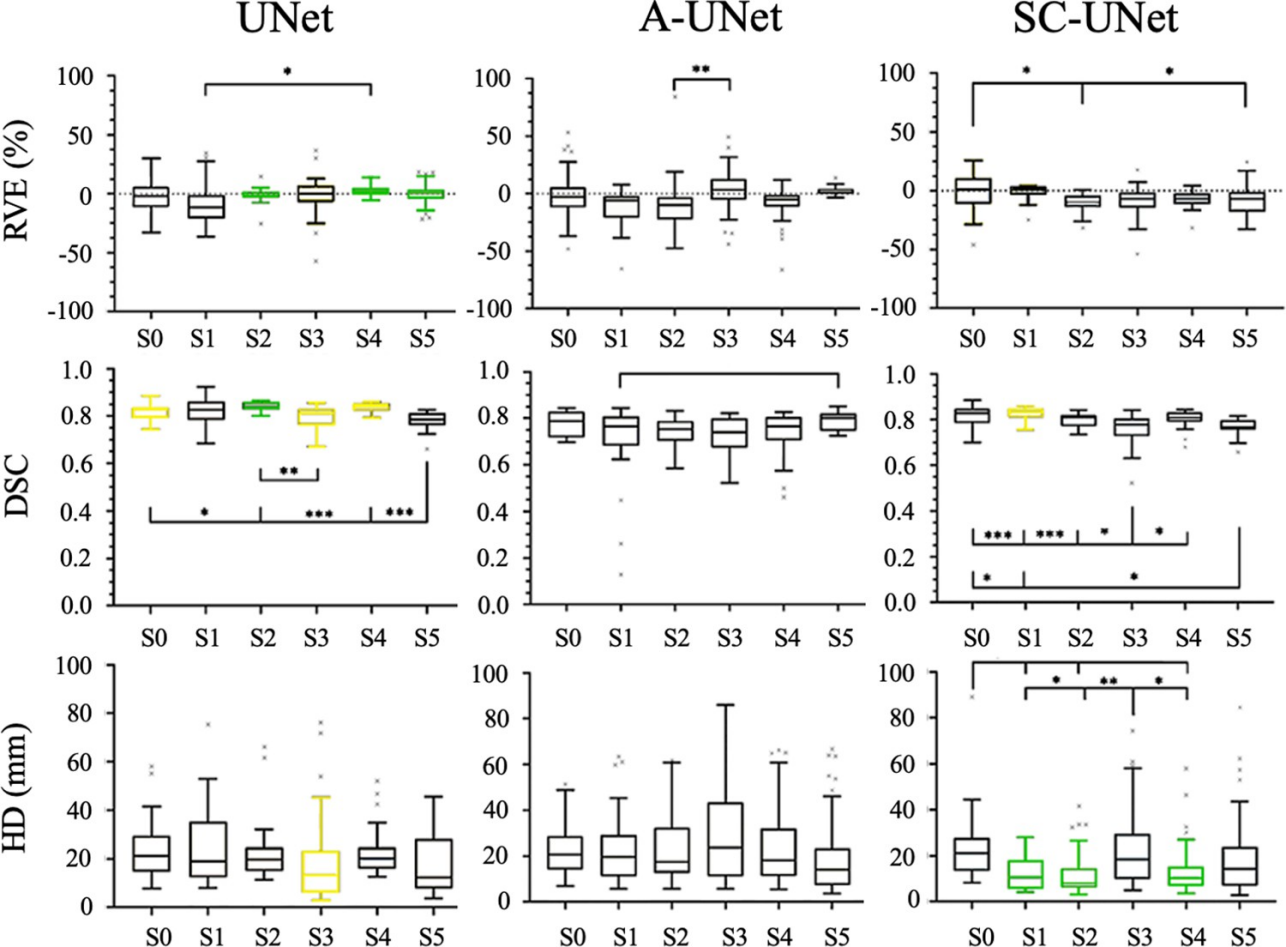
RVE (%), DSC, and HD (mm) calculated from the segmentations of 23 muscles from 6 different testing subjects for the UNet (left column), A-UNet (middle column) and SC-UNet (right column). Connections between distributions highlight significant differences between the mean error of the connected distributions. The boxplots with longer lines pointing at them had significant difference when compared to those with shorter lines pointing at them (* p <0.05, ** p<0.01, *** p<0.001). The three error metrics found for each subject across the three CNN models were also compared. A green boxplot highlights the statistically optimal result for a particular subject with significant improvement from the other two models (p<0.05). A yellow boxplot highlights the statistically best results for a particular subject with significant improvement from one of the other two models (p<0.05). Note that S0 is the initial test subject used in test 2).

The three CNN models with the augmented images included in the training dataset were retrained, exchanging the testing subject for each of the five other subjects. Fig 7 presents the RVE, DSC, and HD found across the three CNN models for each of the six subjects tested.

First, the performance of each of the three models was assessed independently with different testing subjects. The UNet was highly consistent considering the RVE found across the six testing subjects (S) with absolute mean RVE below 2% in most cases (with the exception of S1 for which RVE was ~8%). The only significant difference was found between S1 and S4 (p = 0.017). For DSC, subjects S0 (initial testing subject), S1, S2, and S4 were segmented with mean above 0.80, whereas S3 and S5 were segmented with mean DSC just below 0.80. Significant but small decrease in DSC was found between S2 and S3 (5.5%, p = 0.009). Conversely, the DSC found for S5 was significantly lower than those of S0 (4.1%, p = 0.044), S2 (6.9%, p<0.001), and S4 (6.3%, p<0.001). The HD were consistent across the six subjects, with mean HD between 17.2 mm and 25.4 mm, with no significant differences (p>0.161).

The A-UNet presented mean RVE of between -10.3% and 6.4%, with only S2 being segmented with a significantly lower accuracy than S3 (p = 0.009). The mean DSCs found with A-UNet were between 0.72 and 0.79 across the six testing subjects. S1 was segmented with a significantly lower DSC than S5 (p = 0.032). No other significant differences were found across the six testing subjects for DSC. The mean HD across the six subjects segmented using A-UNet were between 19.7 mm and 29.5 mm, with no significant differences (p>0.158).

The mean RVE found for the SC-UNet were between -0.5% and -10.2%, consistently underestimating the volume of the muscles when compared to the ground truth reference segmentations. The S0 was segmented with a significantly lower RVE than S2 (p = 0.002) and S5 (p = 0.026). Similar to the UNet results, the mean DSC found for S0, S1, S2, and S4 were higher than 0.80, whereas for S3 and S5, the DSC values were slightly lower than 0.80 (0.76 and 0.77, respectively). S3 was segmented with significantly lower DSC than S0 (p<0.001), S2 (p<0.001), and S4 (p = 0.012). S5 was segmented with significantly lower DSC than S0 (p = 0.012) and S1 (p = 0.001). Finally, the mean HD for the SC-UNet were between 12.1 mm and 23.0 mm. The HD found for S0 was significantly greater than S1 (p = 0.031), S2 (p = 0.004), and S4 (p = 0.015). Similarly, S3 was segmented with significantly greater HD than S1 (p = 0.026), S2 (p = 0.003), and S4 (p = 0.012).

Comparing across the three CNN models, UNet segmented the muscles with RVE significantly lower than the other two models for S2 (p<0.001 for both), S4 (p = 0.010 for both), and S5 (p<0.001). No significant differences were found between the RVE for S0, S1 and S3. The UNet segmented 4 out of 6 subjects with DSC significantly higher than those from both other models (S2, p<0.001) or than that for A-UNet (S0, p = 0.016; S3, p = 0.008; S4, p<0.001). The SC-UNet was associated with the DSC significantly greater than the A-UNet for S1 (p = 0.001). Finally, the SC-UNet segmented 3 out of 6 subjects with the lowest HD than those for the other models (S1, p = 0.005; S2, p<0.001; S4, p<0.001). S3 was segmented by UNet with HD significantly lower than A-UNet (p = 0.017), but not significantly lower than the SC-UNet (p = 0.424).

**Fig 8:**
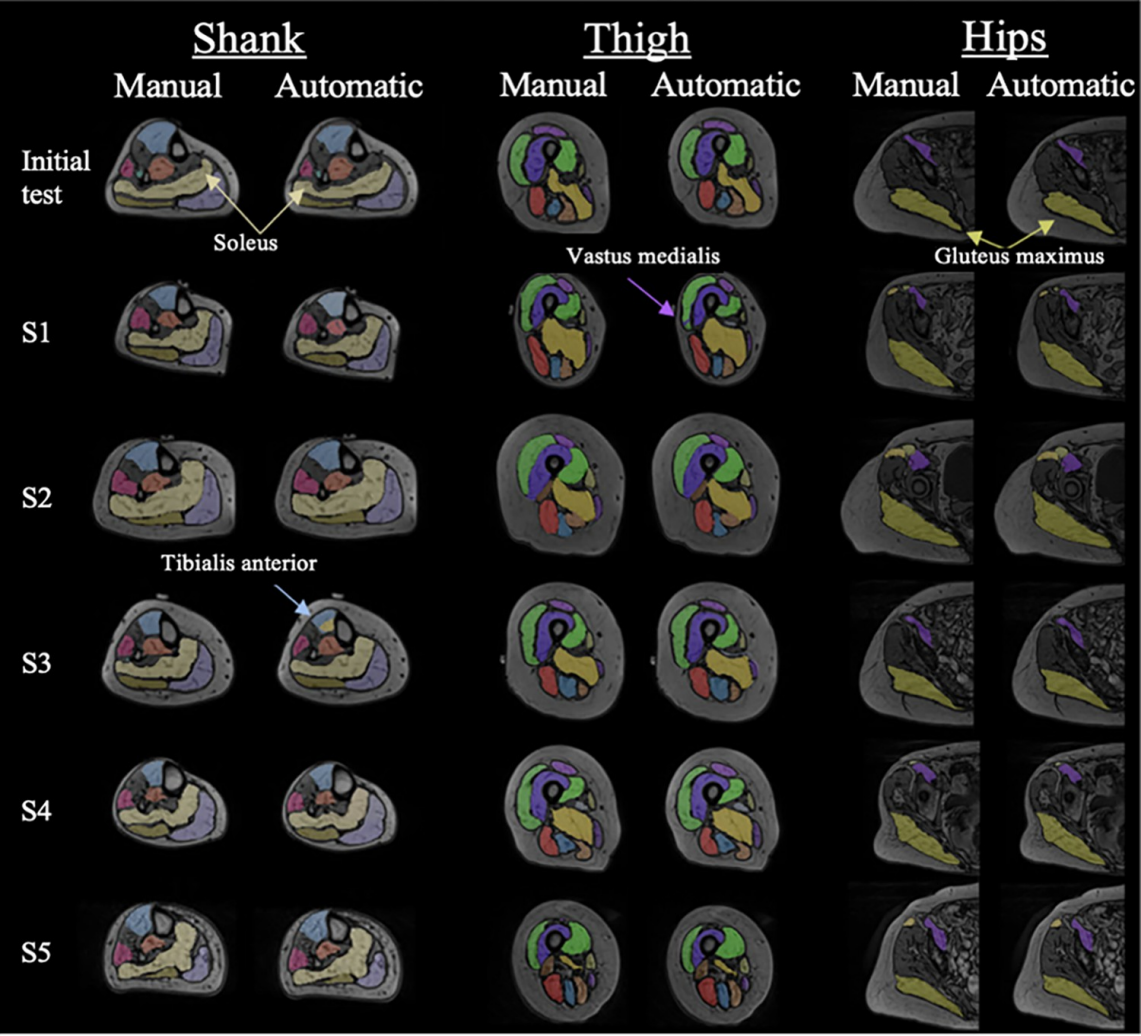
Visual representation of ground truth and automatic segmentations outputted from the best performing model, the UNet trained with the augmented imaging datasets. Three slices taken from halfway along the shank, thigh, and hip sections are presented, with the manual and automatic segmentations shown on the left and right, respectively. Each row of images corresponds to each of the six segmented subjects. Yellow arrows highlight regions that are well segmented, whereas purple and blue arrows highlight regions with segmentation inaccuracies.

In most cases the UNet performed the segmentation with the lowest RVE and highest DSC. Some examples of ground truth reference and automatically generated segmentations are presented in Fig 8. Overall, the segmentations predicted by the UNet were visually very similar to the ground truth segmentations. Both the location and geometry of all 23 muscles assessed were generally well predicted, reflected by the high DSC found, being above 0.85 across all subjects other than the adductor magnus in S3 (DSC = 0.81). The gluteus maximus and soleus (highlighted in Fig 8) were extremely well predicted. However, there were some shortcomings in the predictions, particularly within the shank of S3 (see light blue arrow pointing to tibialis anterior mislabelling, Fig 8) and thigh of S1 (see purple arrow pointing to the vastus medialis mislabelling, Fig 8), where portions of the muscles were clearly mislabelled.

### Muscle by muscle interpretation

**Fig 9:**
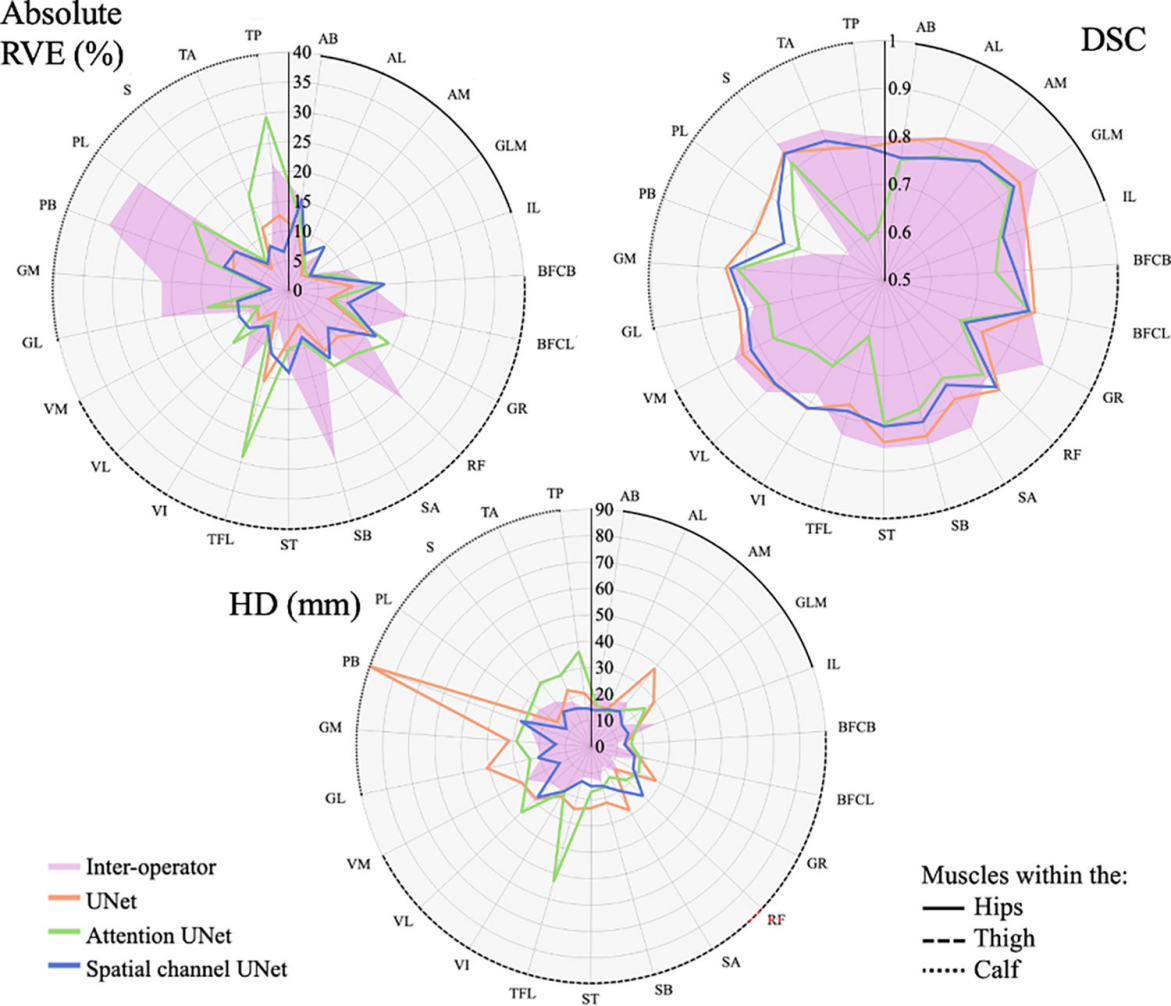
Radar plots showing the absolute RVE (%, upper left hand corner), DSC (upper right hand corner), and HD (mm, lower centre) for the segmentation accuracy found for each muscle, averaged across the 6 tested subjects. The UNet, A-UNet, and SC-UNet appear in orange, green, and blue respectively, with the pink area of the graph representing the errors occurring through inter-operator repeatability. The three locations of the muscles: those within the hips, thigh, and calf, are highlighted with the solid, dashed and dotted black line along the circumference of the radar plots, respectively. The muscles are abbreviated according to Table 1.

Averaging the performance of the three CNN architectures across all 6 tested subjects for each muscle individually, the segmentation accuracy of the three CNN models was comparable (Fig 9). Considering the absolute values of the RVE, on average both the UNet and SC-UNet outperformed the A-UNet for all muscles and particularly for the tensor fascia latae and tibialis muscles. Assessing the DSC, again the UNet and SC-UNet were very similar, but the UNet outperformed the SC-UNet for all muscles (on average) other than the tensor fascia latae and tibialis anterior. On the other hand, SC-UNet was superior to the UNet in terms of the HD, for all but the rectus femoris muscle (Fig 9).

The inter-operator repeatability analyses are also plotted in Fig 9. Comparing the RVE accounted for by the inter-operator variability and the segmentation accuracy of the automatic approaches, the UNet outperformed the inter-operator analysis in the segmentation of 19/23 muscles, the A-UNet for 9/23 muscles, and the SC-UNet for 13/23. The DSC found in the inter-operator analysis was lower than that found using the UNet for 9/23 muscles, the A-UNet for 3/23 muscles, and the SC-UNet for 5/23 muscles. Finally, the HD was greater in the inter-operator analysis in 7/23 muscles for the UNet, 5/23 muscles for the A-UNet, and 12/23 muscles for the SC-UNet.

## Discussion

This study aimed to investigate the capacity of state-of-the-art CNNs to simultaneously segment 23 individual major lower limb muscles from T1-weighted MRI acquired from a cohort of 11 post-menopausal women and proposed novel methods to overcome current computational limitations and data requirements noted within the literature [13,27]. Though similar studies do exist in the literature [2,13,27], these state-of-the-art models have not been tested to segment all major lower limb muscles individually and simultaneously in a cohort with T1-weighted images.

Across three metrics: RVE, DSC, and HD, the 23 muscles were segmented with a moderate to high level of accuracy (minimum, maximum average RVE: -8.6%, 2.9%, average DSC: 0.70, 0.84, average HD: 12.2 mm, 46.5 mm) by all three models tested. All models were shown to have converged through assessment of the training and validation loss curves, suggesting that the models were fully trained and that the training and backpropagation algorithms used were valid. Both the traditional UNet and SC-UNet were able to segment the muscles well within the subject initially chosen for testing (test 2). Upon inclusion of the augmented imaging datasets into the training phase, these networks could segment the muscles with low absolute average RVE below 2.1% and a moderately high DSC of 0.81. The A-UNet [32] was also retrained with the augmented imaging datasets, showing a comparable RVE and a slightly lower average DSC of 0.79. The encoder-decoder module used in A-UNet has been used very effectively in the segmentation of other tissues but has not been used widely in multi-classification segmentation tasks [30,32]. Therefore, the A-UNet may have been negatively affected by the large number of classes (or muscles) in this segmentation task, given that this network was outperformed by the traditional UNet.

The use of augmented imaging data, particularly within a relatively small study cohort, benefitted all three of the models tested, and significantly improved segmentation accuracy in at least one of the three error metrics. The SC-UNet benefitted the most with the inclusion of the augmented database in the training algorithm, implying that it could be improved further with additional data, but even with the augmented database included, it achieved a comparable level of accuracy to the traditional UNet. The spatial channel method was designed to allow the network to understand that fewer muscles could be present within each image slice. The fact that the HD was the lowest on average for the SC-UNet, suggests that this goal was attained. However, the lower DSC found for SC-UNet when compared to the one from the UNet when trained without the augmented dataset suggests that the spatial channel running in parallel with the UNet is not beneficial for capturing individual muscles more accurately. The limiting factor for the SC-UNet could be the high degree of variability present within a small cohort such as this. For example, the testing subject may have a muscle present within a certain image slice at a given percentage along the lower limb, whereas this muscle might not have been present in the other subjects at the same scaled spatial location. In this case, the spatial channel would prevent the inclusion of this muscle in the prediction for that certain image, which could lead to an incorrect segmentation. As is clear from Fig 6, when retrained with additional augmented images, which were shown to increase the variability of muscle structure when comparing the augmented and original datasets [25], this effect was reduced for the SC-UNet. Nevertheless, it remains to be tested if a further augmentation of the dataset would lead to even higher improvement of the evaluation metrics for the SC-UNet. The HD was however lowest when using the SC-UNet, suggesting that the spatial channel enhanced the accurate capture of the surface of the muscles.

The traditional UNet has been used in the past to perform multi-class segmentation of the muscles from MRI data [2,13,27]. The study by Ding et al. [13] segmented two muscle groups and two individual muscles from water and fat suppressed images, inputting two images into the network. The two individual muscles (gracilis and sartorius) that were segmented in the study by Ding et al. [13], were segmented with a DSC of 0.86 on average, around 6% greater than that in this study. However, in this study, 23 individual muscles were segmented with an average DSC of 0.82 across six subjects, slightly lower than that achieved by Ding et al. [13]. The difference between the results presented by Ding et al. and those found in this study could be due to their use of two MRI types as a multi-channel input, where in this study only T1-weighted images were used, bringing less information per training iteration to the network. Zhu et al. [2] used a similar approach, training many different network models to segment muscles from only the calf. Their group presented a method to segment the calf muscles with an average DSC of 0.89 using a hybrid 2D and 3D model, which is significantly greater than that presented in this study of 0.81 DSC on average (considering only the calf muscles). They also tested the traditional UNet, which was able to segment the muscles with an average DSC of 0.87. The main difference between the two studies that may explain the lower DSC in the present study is the input cohort. In fact, in Zhu et al. [2], the study cohort consisted of young subjects both with and without cerebral palsy, in contrast to older post-menopausal subjects in this study. Zhu et al. [2] had a greater number of original subjects (n = 20), which might have enhancing the training of the networks, but the younger subjects used were likely to have a more homogenous muscle tissue appearance in MRI due to the effects of age-related degradation of skeletal muscle tissues [43]. The irregularity of muscle tissues visible within MRI has typically lead to higher operator variability in the manual segmentation process, especially in cohorts with musculoskeletal disorders or older individuals [4,34]. Therefore, it was expected that the automatic segmentations presented in this study were less accurate than those reported by Zhu et al. [2]. In another study, Ni et al. [27] segmented all lower limb muscles using a UNet architecture but exchanged the traditional 2D inputs for full 3D images. They trained 35 individual networks, for a study cohort of 64 young healthy athletes, incurring extreme computational costs and training time. Their study found a segmentation accuracy of approximately 0.90 for DSC, higher than this study and Zhu et al. [2]. This again could be attributed to the higher homogeneity and definition of muscle appearance in the MRI in the cohort of young athletes.

After retraining the three models for the additional five testing subjects, the models were mostly robust to the changes in the testing subject. The segmentations performed with the A-UNet were typically worse than the other two models. The UNet and SC-UNet were generally comparable, with the UNet being superior for capturing the volume of the muscles accurately (lower RVEs), and the SC-UNet being superior for capturing the shape of the muscles (higher DSC). The RVE and DSC were typically slightly better with the UNet, significantly exceeding both other models in a moderate number of subjects (Fig 8). However, the SC-UNet outperformed both other network architectures considering the HD, as shown in Fig 9. Overall, the UNet model was robust to changes in the testing subject, suggesting that if tested for new subjects (not included in the current study cohort), segmentations of similar accuracy might be produced automatically. Additionally, comparing with a previous study using deformable image registration (both single or multi-atlas) to segment the same cohort, the deep learning-based automatic segmentations were more accurate than those obtained in the previous study (RVE = 1.7±20.7%; DSC = 0.73±0.10; HD = 29.7±12.9 mm) [25].

Moreover, the three models segmented the various testing subjects with errors comparable to those incurred in an inter-operator analysis. All three automatic approaches were comparable in their capacity to capture the volume of the muscles, with the A-UNet and SC-UNet able to capture the volume of 39.1% and 56.5% of the muscles more accurately than a second operator, respectively, with the UNet able to segment a large majority of the muscles (82.6%) with lower RVE than in the inter-operator analysis. The UNet was able to segment the most muscles (39.1%) with higher DSC than in the inter-operator analysis, when compared to the other CNN models. Finally, in terms of HD, the SC-UNet captured the surface geometry of the muscles more accurately than in the inter-operator analysis for 52.2% of the muscles, greater than both the UNet (30.4%) and A-UNet (21.7%). In particular the peroneus brevis and longus presented poor repeatability in the inter-operator analysis, but these muscles were segmented with greater accuracy by all three models in terms of both RVE and DSC. These results suggest that deep-learning based models trained using manual segmentation gathered by one operator, might offer more repeatable segmentations than by a second operator for some specific muscles. These results strengthen and highlight the future potential of deep learning-based approaches for automatic segmentations of lower limb muscles from MRI of postmenopausal women [34].

This study has some limitations. First, as is the case with many deep learning applications, the amount of data limited the training phase of all three tested networks. This is clear as the augmented images increased segmentation accuracy across all of them. Future work will generalise the methods explored in this study to incorporate more diverse subjects with varying muscle characteristics (i.e., muscle disorders, athletes, younger and older individuals) and with different scanning parameters (e.g., T2-weighted, Dixon method images). Additionally, the training phase required a significant amount of computational time, which could be boosted with the use of high-performance computers with multiple GPU cores. This was not considered as one of the aims of this study, which was designed to foster the use of these methods without specialised computational resources. Nevertheless, the SC-UNet required a very similar amount of time compared to the traditional UNet, suggesting that other methods that enrich the learning process with meta-data could be adopted with little effect on computational time. Moreover, the 2D approaches used throughout this chapter could limit the learning process as the muscle structures segmented are 3D in reality. Three-dimensional applications of UNet-style structures have been tested in medical image analysis [44], but these approaches typically affect the time and computational requirements on the learning process. Specifically with large images such as these (~250x250x1000 pixels), a 3D application of the UNet would substantially increase the computational expense incurred by the learning process. Additionally, the low number of full 3D labelled images (n = 11) could cause more bias in 3D UNet [41] and lead to reduced overall performance. Comparatively, the number of unique images acquired during the MRI acquisition of this study was much greater than other studies, being between 230 and 323 slices (in 2D), where other studies such as that of Zhu et al. [2], had between 58–94 slices. Therefore, this study had a larger number of images per subject with which to train the various CNNs compared to others, but fewer subjects enrolled in the study. In this study, however, the accuracy of the automatic segmentations produced were lower than that of Zhu et al. [2], suggesting that even with a greater number of images per subject, a greater number of subjects might be required to improve the training and provide segmentations of accuracy comparable to the gold standard, manual segmentation. While in this study data augmentation with virtual subjects was used, a further increase in the number of training subjects could improve the model outputs in future, especially for the models mostly affected by data augmentation (i.e., SC-UNet). Methodologically, the fact that 14 of the muscles were trained to be segmented from the imaging data but were not considered in the analysis of the results could have affected the obtained segmentation accuracy. However, this is necessary as the manual segmentation results were deemed unreliable for those 14 muscles, subject to exclusion criteria described in the section titled Manual segmentation and label generation, which adhered to the current gold standard approach in manual muscle segmentation [4,34]. Including these 14 muscles in the analysis of the results could therefore cause confusion, incorporating segmentations that were found to be nonreproducible. Finally, this study focussed on individual muscle segmentation, in contrast to the majority of previous works that sought to segment the muscle tissue from the other tissues [15,16], typically motivated to assess the level of fat infiltration within the muscle. Future works could focus on optimising the developed algorithms to segment the intra-muscular fat from within individual muscles, as this quantitative measure can be an effective diagnosis strategy for many muscle disorders [15].

## Conclusion

This study showcased the use of two state-of-the-art deep CNNs in the application of multiple muscle segmentation in post-menopausal women as well as a novel CNN that built in an understanding of the spatial location of each image. Though novel deep learning-based methods built to segment the muscles from MRI are still recommended, it was shown that knowledge of the spatial location of the images was beneficial to the novel network’s training in this context. Also assessed was the benefit of training each of the networks with augmented images generated through image registration. It was shown that the accuracy of the predicted segmentations was drastically improved for two out of the three models, but only slightly with the third one. This work highlights the potential of automatic image segmentation methods based on CNNs to automatically estimate geometrical properties of the lower limb muscles from post-menopausal women [45].
